# Amino Acid Metabolomic Profiles in Bovine Mammary Epithelial Cells under Essential Amino Acid Restriction

**DOI:** 10.3390/ani11051334

**Published:** 2021-05-07

**Authors:** Laura López-Diez, Camilo Calle-Velásquez, Mark D. Hanigan, Zulma Tatiana Ruiz-Cortés

**Affiliations:** 1 Research Group Biogénesis, Faculty of Agricultural Sciences, University of Antioquia, Medellín 050034, Colombia; lpzdiez@gmail.com; 2 Faculty of Agricultural Sciences, University of Antioquia, Medellín 050034, Colombia; camicalle@gmail.com; 3 Department of Dairy Science, Virginia Tech, Blacksburg, VA 24061, USA; mhanigan@vt.edu

**Keywords:** abductive analysis, ASNS, bovine, casein, EAA restriction, GOT2, mammary epithelial cell, MAT2A, MDH2

## Abstract

**Simple Summary:**

Cells of the mammary gland obtain their necessary nutrients from the blood to produce milk components, such as casein. To achieve higher productivity, cows are excessively supplemented, thus generating a higher cost of production and affecting the environment. Therefore, this triggers the need for a reduction in the supplementation of essential amino acids without affecting the milk composition. The present in vitro study shows that, through homeostatic and homeorhetic processes, cells have the ability to maintain stable casein levels despite decreasing the percentage of essential amino acids (EAAs) supplied. These findings could contribute to the proposal of more efficient nutritional strategies at lower environmental and economic costs.

**Abstract:**

Mammary epithelial cells (MECs) in culture are a useful model for elucidating mammary gland metabolism and changes that occur under different nutrient disponibility. MECs were exposed to different treatments: 100% EAA for 8 h and 24 h restriction (R); 2% EAA for 8 h and 24 h R; 2% EAA for 8 h and 24 h + 100% EAA for 8 h and 24 h restriction + re-feeding (R + RF). Western blotting and protein quantification was performed. The Kyoto Encyclopedia of Genes and Genomes (KEGG) software identified the amino acids (AAs) and signaling pathways. The chi-squared test, multiple classification analysis, and analysis of variance were used for the purification and identification of data. Intracellular casein levels were not affected. The KEGG analysis revealed that the important pathways of metabolism of AAs, which were involved in processes related to metabolism and biosynthesis of phenylalanine, tyrosine, and tryptophan (fumarate, acetyl-CoA, and tricarboxylic acid (TCA) cycle), were affected by both R and R + RF treatments, mainly through the glutamic-oxaloacetic transaminase-2 enzyme. Additionally, metabolic processes mediated by the mitochondrial malate dehydrogenase, S-adenosylmethionine synthetase, and asparagine synthase proteins positively regulated the carbohydrate pathway, pyruvate, and TCA cycles, as well as the metabolism of alanine, aspartate, and glutamate metabolism (carbohydrate and TCA cycle). We hypothesized that MECs have the capacity to utilize alternative pathways that ensure the availability of substrates for composing milk proteins.

## 1. Introduction

Given the current feeding conditions, where only 30% of nitrogen (N) offered to cattle via diet is metabolized [1], large losses are excreted to the environment, thereby increasing the costs of production. Approximately 20% of the protein offered is eliminated as feces; 15% is eliminated as ammonium from the rumen; 40% is catabolized after absorption; and only 25% is recovered in milk protein [2]. The above-mentioned points reflect the inefficiency of the usage of proteins in lactating cows [3]. In addition, identification of the optimal amino acid (AA) profile of dietary protein for these types of cows is not specified by nutritional models [4]. Therefore, in the field, the levels of raw protein in the diet are usually higher than those required by animals [5].

The relatively low values of dietary protein in the mammary gland suggest inefficiency in the use of N compounds [6]. The capture of AAs by the mammary gland is quite low, with only 22% daily influx of arterial AA [6]. High splanchnic tissue catabolism of AA and its subsequent conversion to excreted urea is a consequence of this low mammary AA capture, which contributes to the low 25% N efficiency in dairy cows [3].

Three strategies have been identified to improve the efficiency of the usage of N in animals and decrease the elimination of N: (1) increase in animal productivity; (2) reduction in excess N in diets; and (3) the provision of specific nutrients in a limited way, for example, by limiting AAs to meet the requirements more precisely [4]. In vitro studies could contribute to the development of the last two strategies, because they are based on a model of bovine mammary epithelial cells (MECs), which are producers of proteins and other milk molecules. Analysis of MECs as a fundamental unit within the lactancy implies an understanding of the cellular mechanisms that self-regulate their functions. Furthermore, it is well known that cell culture provides a useful experimental tool that has many advantages over in vivo research, such as tight control of the chemical and physical environment, higher throughput, testing of a high number of treatments on isolated cells, and reduced animal-use [7].

Recently, Li and Jiang have published the metabolomic profiles in the yak mammary gland during the lactation cycle [8]. They reported the differential metabolites involved in pathways, including AA, biosynthesis of other secondary metabolites, carbohydrate, energy, lipid, cofactors and vitamins, and nucleotide metabolism, which are related to mammary gland development, lactogenesis, and lactation [8]. Studies on MECs regarding their inherent metabolic status under AA challenges, as in vitro model for limited nutrients provision, are scarce; this study can provide evidence to further the knowledge of bovine lactation metabolism.

Thus, the abductive analysis drives us to hypothesize that MECs subjected to a restriction (R) of essential amino acids (EAAs) have the ability to maintain the production of milk proteins, such as casein, which are involved in homeorhetic metabolic processes.

## 2. Materials and Methods

### 2.1. Cell Culture and Treatments

Bovine primary MECs were obtained from the State Key Laboratory of Animal Nutrition, Institute of Animal Science, Chinese Academy of Agriculture Sciences, Beijing, China. Cells were isolated from Chinese Holstein dairy cows [9]. To confirm that the cells were of bovine and epithelial origin, identification of epithelial markers via proteomics (i.e., cytoskeletal 8 (UniProt ID = F1MU12) and cytokeratin 18 (UniPro ID = F6S1QO)) was conducted (data not presented) [10]. Casein synthesis ability was confirmed in a previous experiment [11].

Cells were seeded at 1 × 10^5^ cells/well onto 6-well plates (Corning^®^, Corning, New York, NY, USA) and grown in Dulbecco’s modified Eagle’s medium (DMEM/F-12; Thermo Scientific^®^ Gibco ^®^, Waltham, MO, USA). Glucose and sodium bicarbonate were added at 3.15 g/L and 1.18 g/L, respectively. The medium was supplemented with 50 mg/mL transferrin, 2 mg/mL insulin, 1 mg/mL prolactin, 25 mg/mL progesterone, 100 mg/mL hydrocortisone (Sigma^®^, St. Louis, MO, USA), 0.25 mg/mL epidermal growth factor, 1% glutamine, 1% antibiotic–antimycotic mix (Sigma^®^, St. Louis, MO, USA), and 10% fetal bovine serum (Thermo Scientific^®^ Gibco^®^, Waltham, MO, USA). Cells were cultured for 6 days after confluence (90–100%).

The cultured cells were subjected to different challenges by the implementation of a 2 × 2 × 2 factorial experimental design, where the first factor corresponds to a variation of concentrations of EAA in two levels (2% and 100%) of the complete concentration in DMEM; the second factor is the R time with two levels (8 h and 24 h); while the third factor is the re-feeding (RF) time (8 h and 24 h). The last two factors were related to each other by a restriction + re-feeding time (R + RF), as previously described [10,11,12]. This resulted in a total of seven treatments, which were replicated four times in duplicate: Control-Treatment 1 (T1): 100% EAA; Treatment 2 (T2): 2% EAA for 8 h R; Treatment 3 (T3): 2% EAA for 24 h R; Treatment 4 (T4): 2% EAA for 8 h R + 100% EAA for 8 h RF (R + RF); Treatment 5 (T5): 2% EAA for 8 h R + 100% EAA for 24 h RF (R + RF); Treatment 6 (T6): 2% EAA for 24 h R + 100% EAA for 8 h RF (R + RF); and Treatment 7 (T7): 2% EAA for 24 h R + 100% EAA for 24 h RF (R + RF).

### 2.2. Total Protein Extraction

Cells were washed twice with cold PBS followed by the addition of cold RIPA (Thermo Scientific^®^ Pierce^®^, Waltham, MO, USA) containing three non-ionic and ionic detergents enabling the extraction of membrane, nuclear and cytoplasmic proteins. Protease (Promega^®^, Madison, WI, USA) and phosphatase inhibitor cocktails (Thermo Scientific^®^, Waltham, MO, USA) were also added to prevent proteolysis and to maintain protein phosphorylation. After 5 min of exposure on ice, cells were scraped from the plates, and centrifuged at ~14,000× *g* for 15 min. Supernatants were transferred to a new tube, and total protein concentration was measured in duplicate using bicinchoninic acid (Thermo Scientific^®^ Pierce^®^ BCA, Waltham, MO, USA) at 562 nm. Cell lysate was stored at −20 °C until further analysis.

### 2.3. Casein Immunoblotting

Proteins (40 μg) were separated by SDS-PAGE (12%) and were transferred to polyvinylidenefluoride membranes (Millipore^®^, Burlington, MA, USA). Blots were blocked for 1 h with Odyssey blocking buffer (LI-COR Biosciences^®^, Lincoln, NE, USA) and incubated overnight at 4 °C with primary rabbit and mouse antibodies (1:1000). The antibodies used recognized α-Tubulin (Cell Signaling Technology^®^, Danvers, MA, USA) as well as casein alpha and beta chains (ABCAM^®^, Cambridge, UK). After five washes for 5 min with phosphate-buffered saline containing 0.01% Tween 20 (Bio-Rad Life Science^®^, Hercules, CA, USA), the blots were incubated for 1 h at room temperature with goat anti-rabbit and anti-mouse secondary antibodies (1:10,000) ligated with fluorescent dyes (IRDye 800 CW and IRDye 680, respectively; LI-COR Biosciences^®^, Lincoln, NE, USA). The blots were washed five more times as described above and scanned using an Odyssey Infrared Imaging System (LI-COR Biosciences^®^, Lincoln, NE, USA). The signal intensity of target proteins was quantified with Odyssey application software (version 3.0), and results were standardized as a ratio of casein expression to alpha-tubulin.

### 2.4. Quantitative Proteomics

After lysing the cells, total cellular protein concentration was measured by bicinchoninic acid assay (Pierce^®^, Waltham, MO, USA). Extracts of total protein were subjected to label-free quantitative proteomics based on ion mobility-enhanced data-independent acquisition. The protocol includes liquid chromatography–integration of ion mobility–mass spectrometry (LC-IMS-MS) analysis and quantification (ISOQuant^®^ software, Mainz, Germany) [13,14]. A total of 1183 proteins were present in ≥90% of the samples. These proteins were used for subsequent analysis. The quantified results from the TOP3 ISOQuant^®^ were used.

### 2.5. Bioinformatics

The Kyoto Encyclopedia of Genes and Genomes (KEGG) software was used for metabolic analysis by means of BlastKOALA tools, using the database of the eukaryotes family in the taxonomic group “animals” [15]. Proteins involved in 17 AA metabolic pathways were selected. The other 6 metabolic signaling pathways (ST) were analyzed, because those related to protein synthesis. The number of proteins per metabolic pathway varied according to the treatment.

### 2.6. Statistical Analysis

For each treatment, an abductive analysis was performed to provide an approximation (hypothesis) of the possible metabolic relationship of the proteome in our in vitro model of MEC. Using the KEGG software results, matrix data were created with the absent or present values of the proteins. A *p*-values matrix of chi-squared distances was calculated, and a pictogram was created to eliminate proteins and metabolic pathways that were not significant (*p* > 0.05). A multiple classification analysis (MCA) was used, and the coefficient of determination (*R*^2^) for each metabolic pathway was calculated in order to determine its relevance in the analysis, thus eliminating routes that were not significant to the first dimension or component (protein synthesis). This procedure was performed until there were no metabolic pathways to eliminate. Proteins detected in the first iteration were used for quantitative analysis of variance (ANOVA) to detect differences between the expressions of proteins. The significant proteins (*p* < 0.05) were selected in terms of the homogeneity of variances. A post hoc study determined the difference between significant expressions of proteins relative to controls (T1). FactoMineR of R program [16] and IBM SPSS Statistics 25 (Armonk, New York, NY, USA) were used for the statistical analysis.

## 3. Results

### 3.1. Mammary Epithelial Cells Casein Expression

**Figure 1:**
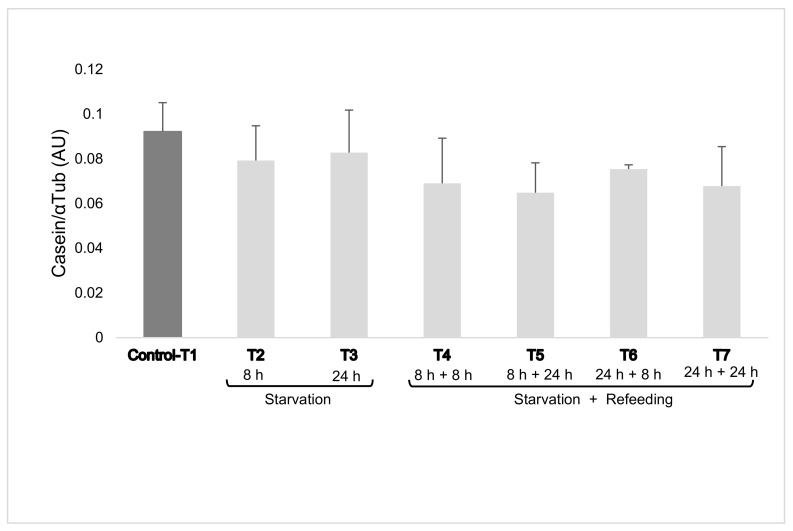
Casein expression in mammary epithelial cells (mean ± SEM). Cells were cultured for six days after confluence. Essential amino acids (EAAs) were added at 100% and at 2% (restriction) of complete Dulbecco’s modified Eagle’s medium concentrations for 8 h and 24 h and re-feeding with 100% EAA for 8 h and 24 h (restriction + re-feeding). The dark column represents cells which were not restricted (Control-T1).

Intracellular casein levels were not affected by treatment with a mean of 0.086 ± 0.02 for the control group (T1) and a mean of 0.081 ± 0.002 and 0.069 ± 0.004 with 2% EAA R (T2–T3) and R + RF (T4–T5–T6–T7) treatments, respectively (Figure 1).

### 3.2. Mammary Epithelial Cell Protein Expression (Proteomics)

A total of 1183 proteins were identified and quantified by LC-IMS-MS. According to treatments, the KEGG software identified between 681 and 892 proteins. AA pathways and ST were selected for analysis, in line with our research focus. Thus, only proteins belonging to 23 metabolic pathways were studied (17 AAs and 6 ST) (Table 1).

In T6, the highest number of proteins (186) was identified, while T7 corresponded to the lowest number of proteins implicated (138) (Table 1).

After chi-squared analysis, only metabolic routes that had a significant relationship with other routes were selected by treatment (*p* < 0.05). This was conducted with respect to the presence of their implicated proteins, while the others were eliminated (Table 2).

Following the selection of significant metabolic pathways, MCA was performed. After the first iteration, proteins that were not in any metabolic pathway were eliminated, with the purpose of only selecting pathways with a minimum of one protein implicated. Finally, the pathways found in all treatments as well as their different related proteins were identified (Table 3).

**Figure 2:**
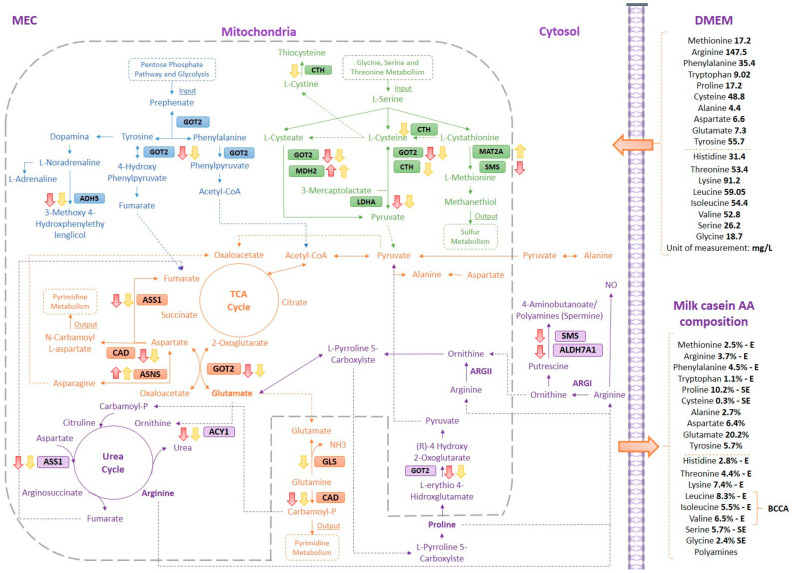
Mammary epithelial cell (MEC) model depicting the interactions between 8 metabolic pathways and the sites of action of the related 13 differential metabolites with significant differences of treatments R (red arrow) and R + RF (yellow arrow) compared to control. In blue, metabolic pathways: (1) Phenylalanine, tyrosine, and tryptophan biosynthesis (top right section); (2) Phenylalanine metabolism (lower right section); (3) Tyrosine metabolism (lower left and middle section). In green: (4) Cysteine and methionine metabolism. In orange: (5) Alanine, aspartate, and glutamate metabolism (middle section); (6) D-glutamine and D-glutamate metabolism (lower middle section). In purple: (7) Arginine biosynthesis (lower left section); (8) Arginine and proline metabolism (lower right section). Essential amino acids (EAAs) in mg/L were added at 2% (restricted) and at 100% (re-fed) of complete Dulbecco’s modified Eagle’s medium concentrations (bold numbers); percentage milk composition of essential (E), semi essential (SE) and non essencial amino acids is indicated [17]. Dotted boxes depict metabolic pathways that receive or originate (output/input) metabolites important from or to the 8 pathways of interest (Modified from [18]).

With the 32 proteins belonging to the 8 metabolic pathways identified in the MCA analysis and casein levels, a database was developed in which the quantification results of each protein were included for each treatment. From these results, ANOVA and post hoc analysis were performed, through which 20 proteins were identified with a significant expression and only 13 were different (*p* < 0.05) with respect to the control (Table 4). Figure 2 illustrates an MEC model, including the 13 proteins and 8 pathways that were implicated.

### 3.3. Metabolic Kyoto Encyclopedia of Genes and Genomes Pathway Analysis

MECs were R and R + RF with EAAs and the protein metabolic pathways were described, including proteins that were differentially expressed (Figure 2). The metabolic pathway of alanine, aspartate, and glutamate metabolism (orange) is the core or center of interactions of other pathways of interest.

#### 3.3.1. Mammary Epithelial Cells under Essential Amino Acids Restricted Treatment

Among the 13 proteins differentially expressed, 8 (L-lactate dehydrogenase (LDHA), spermine synthase (SMS), argininosuccinate synthase (ASS1), carbamoyl-phosphate synthase (CAD), glutamic-oxaloacetic transaminase-2 enzyme (GOT2), aminoacylase (ACY1), aldehyde dehydrogenase family 7 member A1 (ALDH7A1), and S-(hydroxymethyl) glutathione dehydrogenase (ADH5)) suffered from decreased expression under R conditions. They were implicated in relevant pathways, such as pyruvate, polyamines, fumarate, ornithine, pyrimidine, dopamine, acetyl-CoA, urea, and the tricarboxylic acid (TCA) cycle (Figure 2). This suggests a decrease in the use of these mechanisms in MECs as well as the use of alternative mechanisms that resulted in significantly increased expression of two proteins (MDH2 and ASNS) after EAA R (Table 4) compared to the control. MDH2 is implicated in cysteine and methionine metabolism (green), while ASNS is implicated in alanine, aspartate, and glutamate metabolism (orange).

Finally, the expression of three proteins (cystathionine gamma-lyase (CTH), mitochondrial malate dehydrogenase (MAT2A), and glutaminase (GLS)) did not change under R treatment when compared to the control. These proteins were included in cysteine and methionine metabolism, pyruvate metabolism, sulfur metabolism (green), as well as D-glutamine, D-glutamate, and pyrimidine metabolisms (orange), respectively. Thus, these pathways were suggested as not being affected by EAA R (Table 4 and Figure 2).

#### 3.3.2. Mammary Epithelial Cells under Essential Amino Acids Restricted + Re-Fed Treatment

After MEC starvation, cells (after 8 h or 24 h) were RF with DMEM with 100% EAA for 8 h and 24 h. The proteins regulated by these treatments were the same as those affected by the R challenge (LDHA, ASS1, CAD, GOT2, ACY1, and ADH5). The other two proteins (GLS and CTH), which were not regulated by R, also had a reduced expression. Similarly, as in MEC under R, in relation to pyruvate and fumarate pathways, MDH2 and ASNS resulted in increased expression and a third protein not affected by R; MAT2A implicated in sulfur metabolism increased under R + RF (Table 4 and Figure 2). SMS and ALDH7A1 were not affected by R + RF.

In summary, KEGG analysis revealed that the important pathways of the metabolism of AAs, which were involved in processes related to metabolism and the biosynthesis of phenylalanine, tyrosine, and tryptophan (fumarate, acetyl-CoA, and TCA cycle, and dopamine), were affected by both R and R + RF treatments, mainly through the GOT2 enzyme. Additionally, metabolic processes mediated by the MDH2, MAT2A, and ASNS proteins, under the treatments described positively regulated the synthesis of products involved in the metabolism of cysteine and methionine (pyruvate, carbohydrate pathway, TCA cycle, and sulfur metabolism), as well as in the metabolism of alanine, aspartate, and glutamate (fumarate, carbohydrate pathway, and TCA cycle). D-glutamine and D-glutamate metabolism (urea cycle and nucleotides-pyrimidine pathways) was negatively regulated by the treatments.

## 4. Discussion

It has long been known that the concentration of protein in milk is affected by energy supplied in the diet as well as the availability of AA [19].

MEC is a very useful model for elucidating mammary gland metabolism and changes that occur under different nutrient disponibility. The versatility of MEC described by our data provides insights into the strategies used for compensating low EAA availability.

The proposed model (Figure 2) is a summary of abductive and comparative analysis, in which questions regarding the complete set of MEC organelles that give rise to all the metabolic processes of AA biosynthesis and metabolism have been raised. As has been showed, most of these processes occur within the mitochondria; however, there is no evidence of the development of cysteine and methionine metabolism (green) and phenylalanine, tyrosine, and tryptophan biosynthesis and metabolism (blue) within the MEC mitochondria. Our suggestion was made on the basis of participation of GOT2 in these processes, which acts as a mediator of mitochondrial interactions in metabolic pathways, such as alanine, aspartate, and glutamate metabolism (orange).

On the other hand, the presence of MDH2 protein, which acts at the mitochondrial level, would further support the hypothesis with regard to processing of the metabolic pathway of cysteine and methionine (green) inside this organelle (Figure 2). Similarly, we propose to include, at the mitochondrial level, the passage from proline to pyruvate in the metabolic pathway of arginine and proline metabolism (purple), which is also mediated by GOT2.

In relation to phenylalanine, tyrosine, and tryptophan biosynthesis and metabolism, it is important to note that mammary tissues do not require tyrosine to synthesize casein. It is suggested that tyrosine can be synthesized from phenylalanine via the phenylhydroxylase pathway. About 10% of casein-tyrosine can be derived from the hydroxylation of phenylalanine. The mammary gland converts 5–9% of phenylalanine to tyrosine [18].

A recent publication investigated the lactation-related metabolomic profiles in the yak mammary gland using the same bioinformatics methodology of the KEGG software. The authors discussed the clues of metabolic pathways during the lactation cycle that coincide with those that were significant in our study (orange): alanine, aspartate, and glutamate metabolism, TCA cycle, and pyrimidine metabolism [8]. The alanine, aspartate, and glutamate metabolism pathway are considered among the most active pathways during lactation [8], which can be supported by our model because it is the metabolic pathway in which the other significant routes converge. Within this metabolic pathway, the ASNS protein catalyzes the bidirectional passage from aspartate to asparagine. As depicted in Table 4 and Figure 2, this protein presents an increase in its quantification in T3 and T6, which corresponds to 2% of EAA for 24 h R and 2% of EAA for 24 h R + 100% EAA for 8 h RF, respectively. Aspartate is a precursor of many compounds involved in MEC signaling and has also been identified for its participation in the urea cycle and gluconeogenesis in dairy cows. It has been reported that this protein increases when there is a limitation of glucose, asparagine, leucine, isoleucine, histidine, cysteine, glutamine, or a single EAA [20,21], as we have evidenced in the present study. On the other hand, it is important to highlight that a decrease in asparagine can induce cellular apoptosis, which shows the physiological importance of this metabolite in the cell [20,22]. In addition, asparagine is a precursor of oxaloacetate, which has been considered as a limiting compound for the speed of the TCA cycle (gluconeogenesis) [23]. All these put together could indicate that the MEC, stressed caused by R of EAA for 24 h, focuses on the increasing level of proteins, such as ASNS, to optimize cellular gluconeogenesis processes; in this case, through oxaloacetate.

Although 63–84% of aspartate is synthesized from the catabolism of threonine, valine, and isoleucine, which were EAA R to 2% in the present study and normally taken from the medium to support the synthesis of other AAs, we did not find an effect on the cellular integrity during the time of the study or on the synthesis of casein [17,24].

Similarly, this can be reflected in the significant decrease in the ASS1 protein in all treatments, which also interferes with the metabolism of aspartate to fumarate (Figure 2), this being a step prior to the formation of oxaloacetate. We suggest that the route through the ASNS protein is more efficient under fasting conditions [23].

Furthermore, the ASS1 protein catalyzes argininosuccinate formation from citrulline and aspartate, this being a rate-limiting step in the biosynthesis of arginine (purple) in the urea cycle (Figure 2) [25]. Argininosuccinate would later be metabolized to fumarate or arginine according to cellular need, which questions whether it can affect the urea cycle or the net arginine synthesis, an EAA in cows (Figure 2) [18].

In another study, it was observed that the suppression of ASS1 could contribute to a decrease in arginine synthesis and may additionally induce abnormal phosphorylation of Akt, making cells more susceptible to genotoxic stress and leading to cell death; however, this study was performed in astrocytoma cells, non-small cell lung cancer, lung adenocarcinoma cells, and colorectal adenocarcinoma cells [25]. It is believed that the activity of ASS1 in breast tissue is low or null, because ornithine cannot be used for the formation of arginine; MEC takes four-fold more arginine from the medium in order to contribute to the synthesis of proline, ornithine, glutamate, citrulline, aspartate, and urea [26]. In a study conducted with pregnant rats, it was evidenced that, by suppressing arginine from the diet, the development of the mammary gland and synthesis of proteins in it during lactation were affected [18]. However, more recently, a nutrigenomic role of arginine was demonstrated in the control of milk protein synthesis in bovine MECs [19]. Although we showed a decrease in ASS1, there was neither cellular damage associated with this nor an effect on the percentage of arginine that makes up casein [25]. Similarly, low physiological activity of this protein in the mammary gland could explain the non-alteration of arginine metabolism, due to the use of more efficient alternative routes that favor biosynthesis and metabolism of this via precursors, such as proline, which are pathways that were not affected in the present study during any of the treatments in the passage toward arginine [26]. In addition, the foregoing would support the nutrigenomic role of arginine for the synthesis of other milk proteins, but further in vivo studies are needed [18,19].

Apart from representing the center of activity of alanine, aspartate, and glutamate metabolism (orange), the TCA cycle contributes to the synthesis of cellular energy through MEC, and hence, milk production [8,18]. We have evidenced that the MDH2 protein, which showed an increase in all treatments except for T4 (2% EAA 8 h R + 100% EAA 8 h RF), has an action within the TCA cycle (orange) and in cysteine and methionine metabolism (green) (Figure 2). Although the TCA cycle was not a significant metabolic pathway in the present study, its role within alanine, aspartate, and glutamate metabolism (orange) may be crucial for the proper functioning of MEC. The MDH2 protein, which acts in the passage between fumarate and oxaloacetate within this cycle, can contribute to stabilizing the energy metabolism of the cell during the R and R + RF processes [23]. It has been observed in other studies that MDH2 has a higher expression in tissues with high energy demands, specifically for nicotinamide adenine dinucleotide hydrate (NADH). This may be due to the fact that both isoenzymes (cytosolic MDH1 and mitochondrial MDH2) work together in the malate-aspartate shuttle, which helps to transfer the NADH generated in glycolysis to the outside of the mitochondria [27]. It is possible that when performing an EAA R, the MECs focus their activity on glycogenic processes in order to meet their essential productive needs by searching for more efficient alternative energy routes [27]. We propose that the increase in MDH2 protein levels in cysteine and methionine metabolism (green) could indicate that when performing the R and R + RF of EAA, the MECs optimize the processes for the energy metabolism of molecules, such as pyruvate, and thus try to keep the metabolic processes stable.

The increase in MAT2A protein during T7 (2% EAA 24 h R + 100% EAA 24 h RF) in the cysteine and methionine metabolism (green) provides evidence of processes where, despite a prolonged EAA R and a late RF, the MECs have the ability to take advantage of the supplied nutrients to reactivate secondary-homeorhetic processes; in this case, relative to sulfur metabolism (Figure 2). In the cell, sulfur is essential for the structural and metabolic functions of methionine and cysteine, which each have a sulfur atom in their composition [18,28,29]. In addition, these AAs are known to participate in the initiation processes of protein synthesis, structure, and folding in eukaryotic cells, specifically in the synthesis of milk proteins [19,28,29]. The foregoing shows the importance of molecules, such as sulfur, within the composition of semi-essential amino acids (SEAAs) and EAAs (cysteine and methionine, respectively), which, despite representing a low percentage in the composition of casein, play a fundamental role in the synthesis of milk protein. In addition, cysteine and methionine are the only two AAs that contain sulfur and can be incorporated into the protein structure [17,18,28,29]. Another important factor in the action of MAT2A is to promote the formation of l-methionine, which contributes to the synthesis of milk protein [30].

Methionine has been identified as an AA that intensifies the translation processes of mRNA through the formation of the 80S ribosome. It also helps to increase the expression of proteins and activation of STAT5 and mTOR ST in order to regulate increases in milk protein synthesis [19,28]. This reflects the importance of not only sulfur in protein synthesis and cellular metabolism, but the physiological “essentiality” of AAs, such as methionine, for the synthesis of milk proteins [19,28,29]. All of the above would explain the increase in proteins, such as MAT2A, during prolonged cellular stress (such as R of 24 h and a late RF after this 24 h) that we report.

In 2019, Li and Jiang provided evidence that the pyrimidine metabolism pathway is one of the most representative in the lactation cycle of the yak [8]. In the present study, this pathway was not found as being representative during EAA R. However, the metabolic pathways alanine, aspartate, and glutamate metabolism, and D-glutamate and D-glutamine metabolism (orange) interfere through the CAD protein, with the production of metabolites that enter or feed the pyrimidine metabolism (Figure 2). It is known that pyrimidines are essential for the generation of DNA and RNA. In addition, uridine and cytidine contribute to the synthesis of sugar complexes (lactose) and lipid complexes in milk, respectively. Finally, thymine is derived from compounds that can nourish the TCA cycle [22]. CAD decreases its action during treatments with prolonged R (T3: 2% EAA for 24 h R; T5: 2% EAA for 8 h R + 100% EAA for 24 h RF; T6: 2% EAA for 24 h R + 100% EAA for 8 h RF); therefore, we could assume that, in these cases, the cell faces the challenge by maintaining stable energy levels, given the advantage of synthesizing asparagine from aspartate by ASNS, as previously discussed. However, it is important to evaluate the possible impact that this decrease could generate in the metabolism of lactose and fat molecules in milk [8].

The absorption of glutamate and glutamine by the mammary gland is inferior to its secretion in milk, given that most of them are obtained in MECs through the catabolism of threonine, valine, and isoleucine, because they are not EAAs [18,24]. However, in the present study, despite the fact that this AA represents the highest percentage in the composition of casein (Figure 2), we did not find such a decrease when the MEC was R to 2% EAA. This may be due to the fact that the GLS involved in D-glutamine and D-glutamate metabolism (orange) managed to remain stable in most treatments, except in T6 (2% EAA 24 h R + 100% EAA 8 h RF) (Table 4). Indeed, casein concentrations were not affected, which is apparently due to the stability in glutamate synthesis [18,24]. On the other hand, it has been observed that by performing an inhibition of GLS1, the synthesis of asparagine can be reduced because it is a secondary metabolite to glutamine. However, in the present study, during T6, the ASNS protein involved in the synthesis of asparagine increased; we suggest that asparagine levels managed to remain stable despite GLS depletion (Table 4 and Figure 2). In addition, Huang et al. [20] showed that a decrease in GLS1 could affect cell growth, proliferation, and migration in MEC. In the present study, MEC did not demonstrate any of the aforementioned conditions during T6 (data not shown).

The inhibition of proteins such as SMS and ALDH7A1 during the R of EAA (T2: 2% EAA 8 h R and T3: 2% EAA 24 h R) could affect the synthesis of polyamines in milk, which functions on the regulation of lactogenesis and proliferation of the enterocyte in neonates [18]. In the present study, it was shown that despite the R of EAA, when performing the RF, either at 8 h or 24 h, the MEC managed to stabilize the concentrations of both proteins, providing evidence of its great capacity to restore metabolic processes related to lactogenesis by the supply of EAA in a controlled manner [18]. On the other hand, we suggest that, by reducing the action of these two proteins (SMS and ALDH7A1), MECs seek to favor alternative energy routes through the ARGII pathway, which is capable of contributing to the synthesis of pyruvate through proline or the TCA cycle through glutamate.

## 5. Conclusions

This study investigated the AA metabolomics profiles in MEC under EAA restriction in vitro. After the KEGG and statistical analysis, 8 metabolic pathways with 13 proteins were implicated and found to be significantly up- or down-regulated by EAA R and R + RF treatments.

The metabolic pathway of alanine, aspartate, and glutamate metabolism was the core or center of interactions of the other pathways. MEC, under stress conditions, focused on the increase in proteins, such as ASNS and MDH2, in order to optimize cellular gluconeogenesis processes. MECs facing challenged EAA supply seemed to favor alternative energy routes through the ARGII pathway and was implicated in arginine and proline metabolism pathways. Both the urea and pyrimidine pathways were inhibited by EAA treatments. The casein level that did not change significantly under the treatments is an indication of the MECs’ capacity to utilize different strategic pathways to face EAA challenges.

Overall, the pathways described are closely integrated together (carbohydrate, AA, and nucleotide pathways) and play vital roles in regulating lactation function; our results provide a better physiological understanding of the bovine MEC metabolism in vitro, which can help to elucidate the regulated metabolic strategies for lactating cows in the future.

## Figures and Tables

**Table 1:** Number of metabolic pathways and proteins identified per treatment after Kyoto Encyclopedia of Genes and Genomes analysis.

Treatment	KEGG Result ^2^	Metabolic Pathways	Proteins
Control-T1	842 (88.8%)	17 AA + 6 ST ^3^	105 + 67 = 176
T2	809 (89%)	17 AA + 6 ST	115 + 63 = 178
T3	851 (89.2%)	17 AA + 6 ST	115 + 60 = 175
T4	809 (89%)	17 AA + 6 ST	115 + 63 = 178
T5	877 (89.3%)	17 AA + 6 ST	107 + 70 = 177
T6	892 (89.2%)	17 AA + 6 ST	116 + 70 = 186
T7	681 (89.4%)	16 AA + 6 ST	86 + 52 = 138
Total ^1^		23	134

^1^ Different metabolic pathways and proteins implicated. ^2^ Results in percent (%) depict the total number of proteins included in the Kyoto Encyclopedia of Genes and Genomes (KEGG) analysis. ^3^ Amino acid pathways (AA) and signaling pathways (ST).

**Table 2:** Number of metabolic pathways and related proteins identified per treatment after chi-squared test (first iteration; *p* < 0.05).

Treatment	Metabolic Pathways	Proteins
Control-T1	13 AA + 4 ST ^2^	80
T2	13 AA + 4 ST	80
T3	13 AA + 3 ST	79
T4	12 AA + 0 ST	42
T5	14 AA + 4 ST	86
T6	11 AA + 0 ST	47
T7	15 AA + 4 ST	74
Total ^1^	19	111

^1^ Different metabolic pathways and proteins implicated. ^2^ Amino acid pathways (AA) and signaling pathways (ST).

**Table 3:** Number of metabolic pathways and proteins identified per treatment after multiple classification analysis.

Treatment	Metabolic Pathways	Proteins
Control-T1	8 AA ^2^	28
T2	7 AA	22
T3	6 AA	23
T4	6 AA	22
T5	7 AA	27
T6	6 AA	22
T7	6 AA	11
Total ^1^	8 ^3^	32

^1^ Different metabolic pathways and proteins implicated. ^2^ Amino acid pathways (AA). ^3^ The eight metabolic pathways identified were: (1) Phenylalanine, tyrosine, and tryptophan biosynthesis; (2) Phenylalanine metabolism; (3) Tyrosine metabolism; (4) Cysteine and methionine metabolism; (5) Alanine, aspartate, and glutamate metabolism; (6) D-Glutamine and D-glutamate metabolism; (7) Arginine biosynthesis; (8) Arginine and proline metabolism.

**Table 4:** Protein expression variation (up- or down-regulated, arrows) with significant differences (*p* < 0.05) compared to the control (Control-T1: 100% essential amino acids).

KEGG Code	Protein ^1^	Treatments					
		T2	T3	T4	T5	T6	T7
		2% EAA 8 h R ^2^	2% EAA 24 h R	2% EAA 8 h R+ 100% EAA 8 h RF ^3^	2% EAA 8 h R+ 100% EAA 24 h RF	2% EAA 24 h R+ 100% EAA 8 h RF	2% EAA 24 h R + 100% EAA 24 h RF
K00016	LDHA	↓	↓	-	-	↓	-
K00026	MDH2	↑	↑	-	↑	↑	↑
K00789	MAT2A	-	-	-	-	-	↑
K00802	SMS	↓	-	-	-	-	-
K01425	GLS	-	-	-	-	↓	-
K01940	ASS1	↓	↓	↓	↓	↓	↓
K01953	ASNS	-	↑	-	-	↑	-
K11540	CAD	-	↓	-	↓	↓	-
K14455	GOT2	↓	-	-	↓	↓	-
K14677	ACY1	↓	↓	-	-	↓	-
K14085	ALDH7A1	-	↓	-	-	-	-
K00121	ADH5	↓	↓	-	-	↓	-
K01758	CTH	-	-	-	↓	-	-

^1^ L-lactate dehydrogenase (LDHA); mitochondrial malate dehydrogenase (MDH2); S-adenosylmethionine synthetase (MAT2A); Spermine synthase (SMS); Glutaminase (GLS); Argininosuccinate synthase (ASS1); Asparagine synthase (ASNS); Carbamoyl-phosphate synthase (CAD); Mitochondrial aspartate aminotransferase (GOT2); Aminoacylase (ACY1); Aldehyde dehydrogenase family 7 member A1 (ALDH7A1); S-(hydroxymethyl) glutathione dehydrogenase (ADH5); Cystathionine gamma-lyase (CTH). ^2^ Restriction (R). ^3^ Restriction + re-feeding (R + RF).

## Data Availability

Not applicable.
